# Tailoring Natural-Based Oleogels Combining Ethylcellulose and Virgin Coconut Oil

**DOI:** 10.3390/polym14122473

**Published:** 2022-06-17

**Authors:** Simone S. Silva, Luísa C. Rodrigues, Emanuel M. Fernandes, Flávia C. M. Lobo, Joana M. Gomes, Rui L. Reis

**Affiliations:** 1 3B’s Research Group—Biomaterials, Biodegradables and Biomimetics, Headquarters of the European Institute of Excellence on Tissue Engineering and Regenerative Medicine, Ave Park, Zona Industrial da Gandra, University of Minho, 4805-017 Guimarães, Portugal; luisa.rodrigues@i3bs.uminho.pt (L.C.R.); efernandes@i3bs.uminho.pt (E.M.F.); flavialobo@i3bs.uminho.pt (F.C.M.L.); joana.gomes@i3bs.uminho.pt (J.M.G.); rgreis@i3bs.uminho.pt (R.L.R.); 2 ICVS/3B’s—PT Government Associate Laboratory, 4710-057 Guimarães, Portugal

**Keywords:** ethylcellulose, virgin coconut oil, oleogels, oleogelation, 3D archictectures

## Abstract

Oleogels are becoming an attractive research field, since they have recently been shown to be feasible for the food and pharmaceutical sectors and provided some insights into the biomedical area. In this work, edible oleogels were tailored through the combination of ethylcellulose (EC), a gelling agent, with virgin coconut oil (VCO), vegetable oil derived from coconut. The influence of the different EC and VCO ratios on the structural, physical, and thermal properties of the oleogels was studied. All EC/VCO-based oleogels presented a stable network with a viscoelastic nature, adequate structural stability, modulable stiffness, high oil-binding capability, antioxidant activity, and good thermal stability, evidencing the EC and VCO’s good compatibility.

## 1. Introduction

The development of oleogels is becoming an active area of research, since they have recently been feasible for the food and pharmaceutical industry and, in extension, for the biomedical area [1,2,3,4,5]. Oleogels are systems containing self-assembled structures entrapping a vegetable oil in a 3D network [5,6]. These gels are very versatile, as their properties can be tailored by altering either the oil component or the gelling agent, or even the proportion between them. The essence of producing oleogels is the choice of proper gelators with a low amount, typically less than 10%; however, depending on the desired consistency of the final materials and target application, the concentration of the gelators could be higher [5]. Studies involving oleogel formation have increased due to the possible application as edible food components and as carriers for water-insoluble bioactive substances [5,7,8]. Oleogels formation allows high concentration liquid oil (>90%) to be structured into a “gel-like” system [9]. The resulting products have tailorable textural and oxidative properties depending on their composition. Many gelator molecules, e.g., hydroxylated fatty acids, lecithin, ethylcellulose (EC), and waxes, have been identified for oleogel formation [3,10]. In particular, EC, a cellulose derivative, has been used as organogelator, meaning that it can be directly dispersed in oil above its glass transition temperature. Moreover, EC is commercially available and is also considered food grade, depending on the country [11]. Additionally, it is biocompatible and suitable for use in pharmaceutics [12]. EC has various properties such as non-toxicity, film-forming ability, thermoplasticity, considerable mechanical strength, and toughness [11,13]. Furthermore, EC undergoes a thermoreversible sol–gel transition that, in the presence of liquid oil, allows the oleogel formation [10,13,14]. This behavior is based on the polar entities’ ability to interact with the EC network in the oil phase. These interactions are strongly affected by temperature, solvent, viscosity, oil type, or polymer concentration, which in turn tailor its final gel properties [13,14]. For instance, EC/olive oil oleogels were developed, and the results showed that the oil composition plays a key role in effect the strength of the gel due to its influence on solvent-polymer interactions [10].

On the other hand, Coconut (Cocos nucifera Linn) is a versatile and renewable resource [1]. Its different parts, such as oil, husk, fiber, and water, have been used for industrial and domestic purposes [1,15]. In particular, virgin coconut oil (VCO) can be extracted using a direct pressing technique without heat and chemical refining [16]. It is composed of almost 90–95% saturated fatty acids [17]; its primary fatty acid is lauric acid, which is present at approximately 45–53% [17,18]. Moreover, VCO is a unique vegetable oil with good antibacterial, analgesic, antioxidant, and anti-inflammatory properties, which confer its health benefits [15,19]. VCO’s health benefits have established its role mainly in the food, cosmetic and pharmaceutical industry, as a functional food oil, in the preparation of ice creams, as a component in skin and hair care products, and as a vehicle for drug development [16,20,21]. Compared to those fields, the investigation of VCO in the biomedical area is still in its infancy. However, promising findings suggested an open window of opportunities [4,22,23]. Preliminary studies involving EC and β-sitosterol as a structuring agent in coconut oil on digestion have been investigated [24,25]; however, their preparation and physical properties have not been analyzed. The current study hypothesized that oleogels as 3D networks could be tailored by combining edible components, namely EC and VCO, that could be useful in biomedical applications such as delivery systems or topical approaches. To achieve these objectives, EC/VCO oleogels were produced using different EC and VCO contents and setting temperatures as pathways to tune the gel characteristics, namely micro/macrostructure, viscoelastic nature, stability, and thermal behavior.

## 2. Materials and Methods

### 2.1. Materials

Ethylcellulose (EC), with a viscosity of 46 cP in 5% (*w*/*v*) in an 80:20 solution of toluene/ethanol and 48.9% ethoxy content, was purchased in Sigma Aldrich (St. Louis, MO, USA), and it was used without purification. Virgin coconut oil (VCO), a commercial product, was purchased in Copra Indústria Alimentícia Ltda., Maceió, Brasil. The composition of VCO main saturated fatty acids and their percentual presence (%) was provided by the manufacturer as follows: Lauric acid, C12–43.5%; Myristic acid, C14–18.4%; Palmitic acid, C16–10.3%; Oleic acid, C18–8.6%; Caprylic acid, C8–6.8%; Stearic acid, C18–2.7%; Capric acid, C10–5.4% and Caproic acid, C2–0.5%. In addition, Nile red dye (9-(diethylamino)benzo[a]phenoxazin-5(5H)-one) was purchased from Sigma-Aldrich Pty. Ltd. (St. Louis, MO, USA). All other chemicals were reagent grade and used as received.

### 2.2. Ethylcellulose/Virgin Coconut Oil Oleogels Formation

**Scheme 1:**
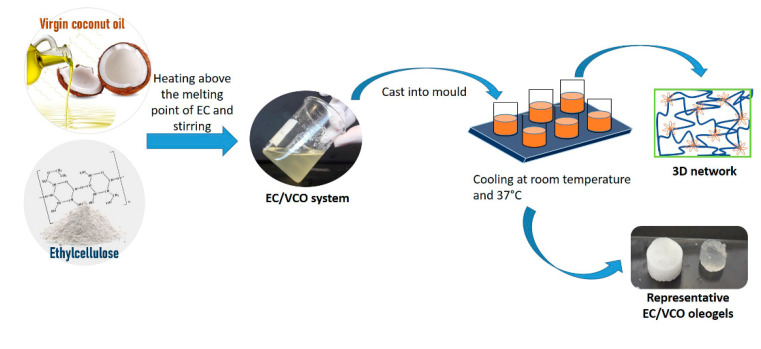
Preparation of ethylcellulose/virgin coconut oil-based oleogels.

Ethylcellulose/virgin coconut oil (EC/VCO) oleogels were produced by dispersing the EC powder on VCO, and the system was heated at 170 °C (above the EC glass transition temperature) in an oil bath coupled with a thermometer. Different contents of EC (5, 10, and 15% *w*/*w*) and VCO (95, 90, and 85% *w*/*w*, respectively) were used, as indicated in Table 1. The system was kept under stirring at 600 rpm for 1 h until a mixture formed. Then, the temperature of the system was reduced to 130 °C and the stirring to 200 rpm. Further, the oleogels were molded into Petri dishes and cylindrical molds (diameter 6 mm), as summarized in Scheme 1. After the oleogel cooled down, samples were kept at room temperature (RT, 22 ± 2 °C) or 37 °C for 48 h. After that, the samples were removed from their molds and stored in plastic bags under a vacuum to avoid humidity absorption. The identification and a summary of the experimental conditions employed on the oleogels development are shown in Table 1.

### 2.3. Rheology

A Malvern Instruments Kinexus rheometer equipped with a stainless steel parallel-plate sample holder 8 mm in diameter and between 5 and 7 mm gap (according to oleogel height) was used to perform the oscillatory rheological measurements on the oleogels samples. A fixed temperature of 25 °C was used in the experiments. Oscillatory experiments on the samples were preceded by strain sweep curves to determine their linear viscoelastic region (LVR). Strain sweeps were then performed on each sample, acquiring 10 points per decade, from 0.01% to 100% at a fixed frequency of 1 Hz. Mechanical spectra were achieved, and the elastic and viscous modulus (G′ and G″, respectively) as a function of frequency, ranging from 0.01 Hz to 100 Hz, and a constant 0.1% strain. All the tests were performed at least in triplicate.

### 2.4. Oil Binding Capability

The oil-binding capability (OBC) of oleogels was measured according to the centrifuge method [26]. Briefly, 1 g of each oleogel sample was carefully weighted in a previously weighted Eppendorf. The Eppendorfs were left overnight at 37 °C. After that, the Eppendorfs were centrifuged at 10,000 rpm for 30 min at room temperature using a mini centrifuge (Scanspeed, Denmark) to express the oil. Subsequently, the samples’ expressed oil was separated by inversion and drainage for 2 h. After, the Eppendorfs were weighted once again (*C*). Equation (1) was used to calculate the OBC, considering the percentage of oil released from the samples after centrifugation.

(1) $$\mathrm{Released}\;\mathrm{oil}\;(\%)=\frac{\left[\left(B-A\right)-\left(C-A\right)\right]}{\left(B-A\right)}\;\times \;100$$

OBC (%) = 100-Released oil (%)

where (*A*) is the weight of the Eppendorf tube before the addition of the sample, (*B*) weight after the addition of the sample, and (*C*) weight after the centrifugation step and excess oil removal.

### 2.5. Free Fatty Acids Determination

Free fatty acids of the EC/VCO oleogels were determined using a Free Fatty Acid Quantification kit (AB65341, Abcam, Cambridge, UK), according to the manufacturer’s instructions. The methyl esters of the fatty acids were prepared according to the method previously described [16] with some modifications. Briefly, 10 mL of n-Hexane was poured into a centrifuge tube, and 2 g of sample was added. The whole mix was shaken for 30 s using a vortex mixer (VELP-ZX3, Italy). This procedure was repeated 2 or 3 additional times along the free fatty acids extraction. After 2 h, one hundred microliters of 2 N potassium hydroxide (KOH) (prepared in methyl alcohol) was added. The reaction mixture was shaken for 30 s by a vortex mixer and further centrifuged at 4000 rpm for 5 min. After centrifugation, 50 µL of each sample supernatant was transferred to each well of a 96 well plate, where 2 µL of the Acyl-CoA Synthetase Reagent was added. The mixtures were mixed and incubated for 30 min at 37 °C. After that 50 µL of the reaction mix (composed of Assay buffer, fatty acid probe, enzyme mix, and enhancer, prepared according to the supplier’s instructions) were added to each well and incubated for another 30 min at 37 °C. Free fatty acid concentrations were measured using a colorimetric methodology by absorbance assessment at 570 nm.

### 2.6. Fourier Transform Infrared Spectroscopy

The chemical characterization was performed by Fourier transformed infrared (FTIR) spectroscopy of the samples performed in a Nicolet 6700 Spectrometer using an ATR mode. The spectrum was collected at room temperature by averaging 32 scans with a resolution of 4 cm^−1^, corresponding to the 4000–400 cm^−1^ spectra region.

### 2.7. Oil Migration Ability and Stability Tests

#### Oil Migration Ability

To characterize oil migration, the amount of oil that each sample lost to filter papers (Whatman #2, 425 mm diameter) was determined independently for each studied time, t, by weighing filter papers before and after the incubation at 37 °C for up to 28 days. Briefly, the clean filter was weighed first, then the gel was set on the filter paper and weighed again, followed by incubation at 37 °C. Oil migration was then calculated as the difference between the oil-impregnated paper weight and the clean paper’s weight, normalized by the oil-impregnated paper weight (Equation (2)). Finally, three replicates were performed for each formulation.

(2) $$\mathrm{Oil}\;\mathrm{migration}=\frac{\left[Wp-Wb\right]}{Wp}\;\times \;100$$

where *Wp* is the paper weight, and *Wb* is the “blank” filter paper weight.

### 2.8. Stability Assays

Stability assays of oleogels were performed at 37 °C up to 28 days, in 15 mL dissolution containers with screw caps, under stirring at 100 rpm. The oleogels were immersed in buffers of pH 7.4 (phosphate-buffered solution—PBS) and pH 5 (citric acid/sodium phosphate buffer, prepared using a 0.1 M citric acid monohydrate (C_6_H_8_O_7_·H_2_O, with MW 210.14 g/mol) solution and a 0.1 M trisodium citrate dihydrate (C_6_H_5_O_7_Na_3_·2H_2_O, with MW 294.12 g/mol) solution). According to Dawson et al., to prepare 100 mL of pH 5 buffer solution, 35 mL of the acid solution was mixed with 65 mL of the citrate one [27]. The solutions were changed each week to keep their stability and activity. The weight loss was determined by using Equation (3):

Weight loss = [(W_o_ − W_f_)/W_o_] × 100 (3)

where W_o_ represents the initial dry mass, and W_t_ the final dry mass.

### 2.9. Antioxidant Activity

The antioxidant activities of the EC/VCO-based oleogels were determined using the 2,2-diphenyl-1-picrylhydrazyl (DPPH) radical scavenging assay [28]. For that, the samples were put in n-hexane (10 mL of n-hexane for 2 g of sample) for 4 h to guarantee the VCO dissolution from the samples. A solution of gallic acid (0.5%) prepared in osmotized water was used as a control. The systems were subjected to different shaking cycles of 30 s during the 4 h. Further, 100 µL of KOH 2N (previously prepared in methanol) was added to the system, which was shaken for another 30 s. The systems were then centrifuged at 200 rpm for 5 min, and after that, the supernatant was separated and stored at RT until use. For DPPH determination, a 1 mM solution of DPPH in ethanol was prepared, and 4 mL of this solution was added to 4 mL of each sample supernatant. These solutions were thoroughly vortexed and incubated in the dark between 1 h and 24 h. After each time point, 100 µL of the mixture was transferred to each well of a 96-well plate. The absorbance was measured at 517 nm against a blank sample lacking a scavenger in a 96-well microplate reader (SpectraMax i3x, Molecular Devices, San Jose, CA, USA). The antioxidant capacity was calculated by using the following Equation (4):

(4) $$\mathrm{AA}\;(\%)=\frac{A0-A1\;\;}{A0}\;\times \;100$$

where *A*0 is the absorbance of the control (DPPH solution alone) and *A*1 is the absorbance of the sample.

### 2.10. Differential Scanning Calorimetry

The thermal behavior of the samples was determined by differential scanning calorimetry (DSC) in TA Instruments DSC Q100 equipment. The DSC analysis was performed using 6 mg of each sample under a nitrogen atmosphere (flow rate of 50 mL·min^−1^) and a heating rate of 10 °C·min^−1^ for assay. Two thermal cycles were performed, the first cycle from −40 °C up to 165 °C intending to erase the thermal story of the sample, and the second from −40 up to 250 °C. Three replicates of each condition were performed. From the thermogram analysis, it was determined the melting temperature (T_m_) and crystallization temperature (T_c_), as well as the respective melting (ΔH_m_) and crystallization (ΔH_c_) enthalpies.

### 2.11. Statistical Analysis

All the performed tests were conducted in triplicate, and the obtained data points are presented as mean values and standard deviations. Statistical analysis was performed while using the GraphPad Prism 8.0 software (GraphPad Software, San Diego, http://www.graphpad.com). The data were analyzed using Tukey’s multiple comparisons test to determine statistical differences. The significance levels between the groups were set for * *p* < 0.05, ** *p* < 0.01, ***p < 0.005 and **** *p* < 0.0001. The data were presented as mean ± standard deviation (SD).

## 3. Results and Discussion

EC/VCO-based oleogels were produced by dispersion EC into VCO with appropriate processing that involved heating at a temperature above the glass transition temperature of EC, which occurs at 140 °C [14], stirring, and then set upon cooling and molding under RT or 37 °C. During this processing procedure, the EC was required to dissolve VCO and form an intricate solid network that entraps the oil [10,29]. At low temperatures (below 25 °C), VCO is solid, slow to oxidize, has a long shelf-life, and a smoke point or burning point, i.e., the temperature at which oil stops shimmering and starts smoking, relatively high: between 177 °C and 190 °C, which allows it to support high temperatures during processing. This last feature is an advantage for the oleogelation process. The temperature measured inside the EC/VCO mixture was about 160 °C, and the EC/VCO mixture initially had a white color but became yellow and clean, as the EC particles were no longer visible (see Scheme 1). Previous studies [10,30,31] reported the ability to change the EC oleogel properties using different strategies such as temperature manipulation during dissolution, setting, storage, oil polarity, and the addition of surfactant. Our preliminary studies (data not shown) indicated that an increase in the EC content above the 15% *w*/*w* promotes the formation of oleogels solutions with high viscosity, which implies difficulties in their manipulation. Therefore, the EC and VCO ratios from 5–15% (EC) and 95–85% (VCO) were used to tune the gel characteristics of the oleogels regarding their viscoelastic nature, structure, stability, and thermal behavior. Furthermore, similar experiments were performed using almond oil and EC, but the resulting materials were not comparable to obtained features in EC/VCO-based oleogels.

**Figure 1:**
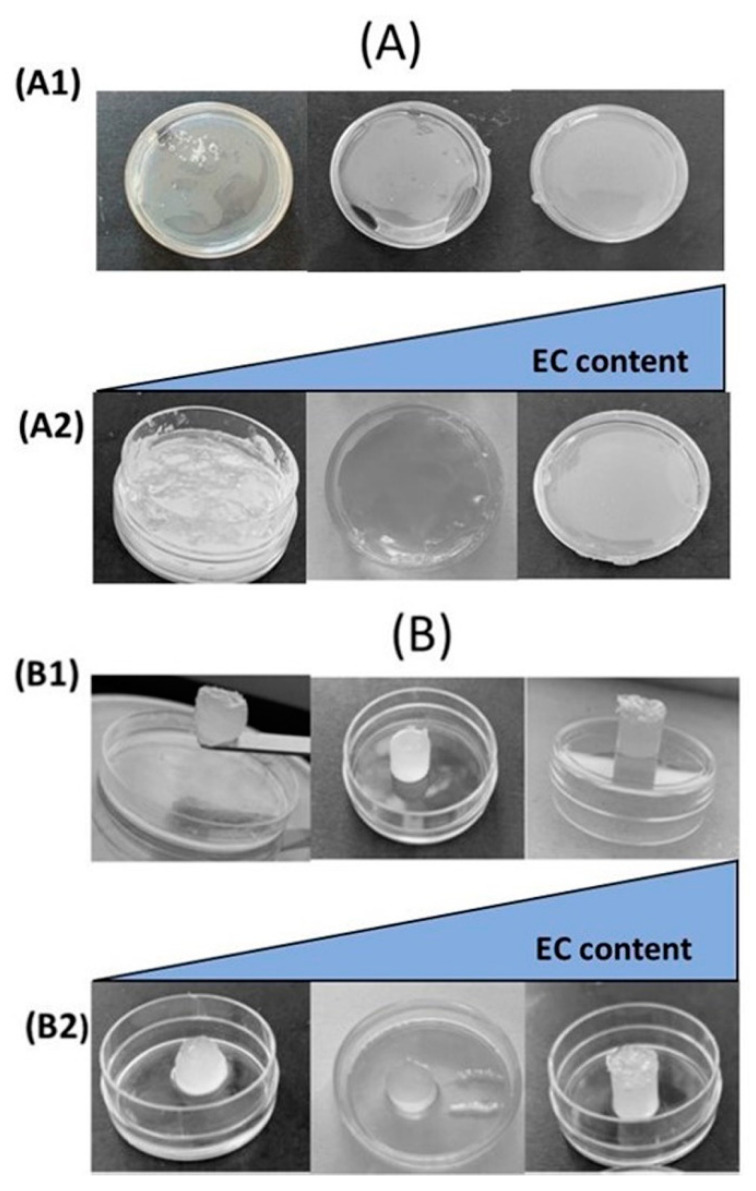
Macroscopic visual appearance of the EC/VCO-based oleogels prepared at different EC/VCO content (**A**) before molding—RT (**A1**) and 37 °C (**A2**); and (**B**) after molding—RT (**B1**) and 37 °C (**B2**).

Considering that the molding temperature of the oleogels could influence their properties and behavior [1], the molded EC/VCO samples were allowed to cool and set at RT or 37 °C. Under macroscopic visualization (Figure 1), we can notice that the EC/VCO-based oleogels have good homogeneity in all conditions. Moreover, the findings showed few differences in the consistency of the oleogels after setting at 37 °C. However, EC/VCO-based oleogels with 5% of EC presented a gel-like behavior, becoming stiffer with the EC concentration increase, as expected due to the increase in the polymeric structural support. Similar behavior was also observed for samples prepared at RT.

### 3.1. Rheology

**Figure 2:**
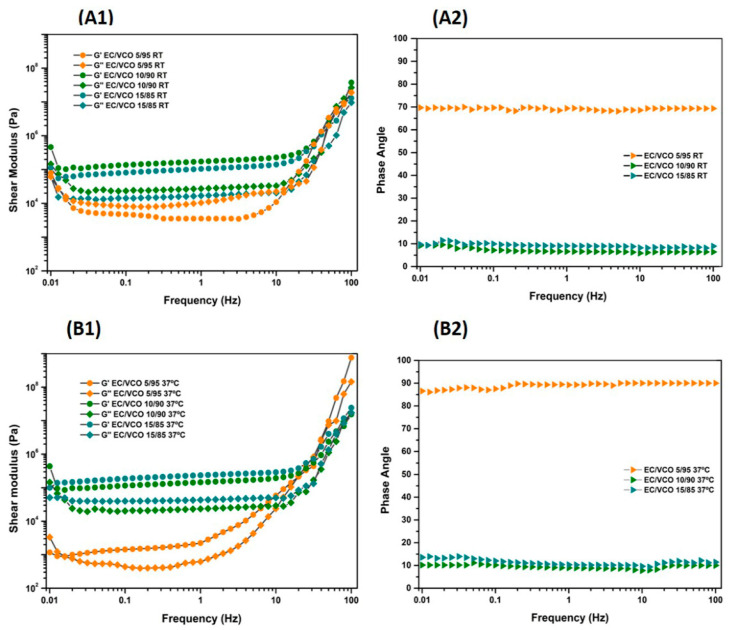
Viscoelastic properties of the molded EC-based (**A1**,**B1**)/VCO-based (**A2**,**B2**) oleogels in the function of the frequency, grouped according to the molding temperature: (**A**) RT and (**B**) 37 °C.

In oleogels, the oils are changed by molecular interactions with organogelators, which results in a decrease in fluidity and rheological features of solid fats. Thus, the oleogels have an oil liquid phase but possess a solid-like behavior. Therefore, the rheological properties of oleogels are important clues to evaluate their behavior during processing, storage, and application, which could have high impacts on food, pharmaceutical, and biomedical approaches. Therefore, the storage, G′, and loss, G″, moduli as a function of the frequency for the different molded EC/VCO oleogels were acquired (Figure 2, Table 2. These data concluded that most of the prepared formulations showed a solid-like behavior because G′ values were always higher than G″ values at all frequency regions, similar to those reported for other oleogel systems.

The rheological studies on oleogels showed that both G′ and G″ increased with the frequency, confirming the samples’ elastic nature. Changes in temperature during oleogel molding and the EC content increase in the formulations could influence the rheological features of the structures. For instance, higher molding temperature (37 °C) on oleogels could imply higher molecular mobility of the VCO molecules, which could increase their interaction with EC molecules.

The loss factor (tanδ value), i.e., the ratio of G″/G′, defines gel’s strength. For example, if a sample or condition holds the G″/G′ ≤ 0.1, it would be accepted as a strong gel [32]. The loss factor of the samples within the measured frequency range was between 0.15–0.71 (Table 2), indicating that almost all the EC/VCO-based oleogels present a gel-like behavior varying from soft to almost strong gels. Still, none satisfied the rheological requirements of a “strong gel”, the strength of which is directly correlated with the EC concentration on the oleogel formulation. The only exception observed was for the composition 5/95_RT was G″ > G′, which is correlated to a viscous sol [33]. The distinct behaviors noted between the different molding temperatures for the 5/95 oleogel formulations, soft gel for 37 °C, and viscous sol for RT, suggest that the RT molding led to a less organized structure and a reduced interaction of the oil molecules with the polymeric network. In other studies [34], the effect of reducing the interactions among EC chains by forming hydrogen bonds in ethylcellulose–monoglyceride–candelilla wax oleogel modified their rheological behavior.

Similar to earlier reported data [35,36,37], the increase in polymeric matrix content in the oleogels leads to an increase of G′ and G″ values with increasing frequency, suggesting the formation of intra- and inter-molecular hydrogen bonding with and between EC polymeric chains [36]. This fact suggests that the interface gel network can provide a mechanical barrier and increase the emulsifying stability of the system [35].

### 3.2. Oil Binding Capability

**Figure 3:**
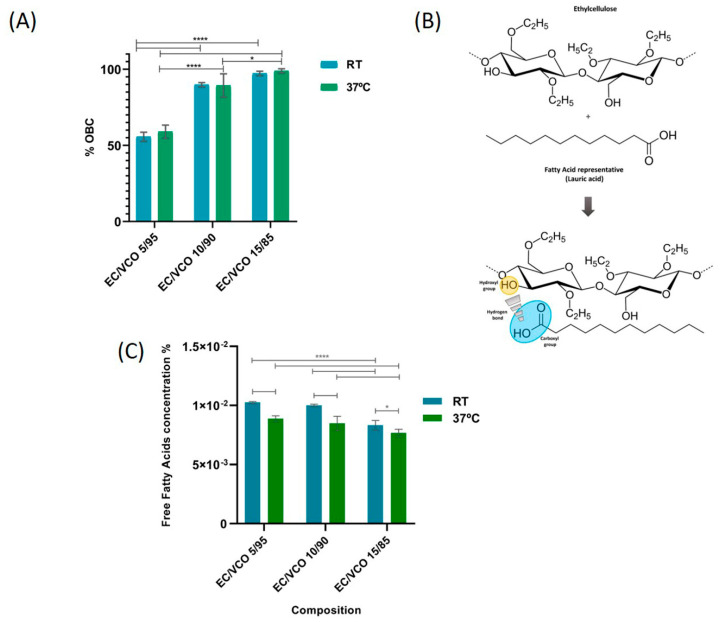
(**A**) Oil binding capacity (OBC) of EC/VCO oleogels with different contents of EC and VCO, and molded setting temperature: RT and 37 °C (**** *p* < 0.0001 and * *p* < 0.05); (**B**) representative scheme of the possible formation of hydrogen bonds established between EC and lauric acid (VCO) molecules; (**C**) fatty acids quantification of the EC/VCO-based oleogels (**** *p* < 0.0001 and * *p* < 0.05).

Oleogel capability for holding the oil is one of the most important features for evaluating gel structure [38]. The oil binding capability (OBC) is influenced by the interactions between oil and gelator molecules in the oleogel [39]. Figure 3A showed an apparent increase in OBC (up to 98%) with EC content in EC/VCO oleogels. Moreover, the EC content influences the OBC ability significantly more than the molding temperature. Then, we hypothesized that the EC/VCO oleogel formation could be driven by hydrogen bonds between the carboxyl head group of the lauric acid and free hydroxyl groups on the EC backbone. In that sense, the increase in EC until 15% (*w*/*w*) into the oleogel formulation can imply more polymer/oil interactions, explaining the higher OBC values and, therefore, the greater ability of those formulations to retain VCO. The obtained OBC values for EC/VCO oleogels were similar to the previous studies on EC/rapeseed-oil-based oleogels [40], where an increase in the EC concentration implied an increase in the OBC values (from 77 to 96%). Figure 3B represents the possible interactions between VCO (lauric acid) and EC. A similar mechanism was suggested by Eisa et al. [41], which described that as the system cools below 100 °C, the dispersed EC molecules become stabilized by forming hydrogen bonds between EC strands, creating a polymeric network that entraps the oil phase and provides mechanical support.

The quantification of free fatty acids on EC/VCO oleogels showed a decrease in the % free fatty acids in the EC/VCO oleogels with higher EC content (Figure 3C). Those findings align with the hypothesis described above, where the increase in hydrogen bonds in the oleogels implies a reduction in the amount of free fatty acids that are able to migrate to the surrounding media.

### 3.3. Structural Features

The structural differences that occurred in the developed EC/VCO-based oleogels and their pure components (EC and VCO) were analyzed by FTIR (see Supplementary Information Figure S1). In the EC spectrum, the characteristic bands at 2967 cm^−1^ and 2868 cm^−1^ were observed due to C-H stretching vibration, and another peak at 1061 cm^−1^ corresponded to –C–O–C stretching [42]. Considering the VCO spectrum, the characteristic bands occurs at 2916 cm^−1^ (–CH_3_ asymmetrical stretch), 2915 and 2846 cm^−1^ (symmetrical and asymmetrical stretching of –CH_2_), 1741 cm^−1^ (–C=O stretch), 1474 cm^−1^ (–CH_2_ bending), 1150 cm^−1^ (–C–O stretch; –CH_2_ bending), 1109 cm^−1^ (–C–O stretch), 1061 cm^−1^ (–C–O stretch) and 730 cm^−1^ (*cis*-CH CH–bending out of plane) [43].

The EC/VCO spectra of oleogels looked very similar and showed the typical absorption bands for EC and VCO [42,43]. In fact, as VCO was the principal component (ranging its ratio from 85 to 95%) in the formulations, its characteristic bands dominated the FTIR of the oleogels systems. A band shift at 1150 cm^−1^ (–CH_2_, VCO) to higher wavenumbers (1165 cm^−1^) occurred in the EC/VCO spectra compared to the VCO spectrum, changes in the VCO fatty acids [43], suggesting the establishment of interactions of them with the polymer into the oleogel network. Furthermore, other studies have demonstrated that EC in vegetable oils develops supramolecular structures mainly as a result of hydrogen bonding among EC chains [29,44].

### 3.4. Oil Migration

**Figure 4:**
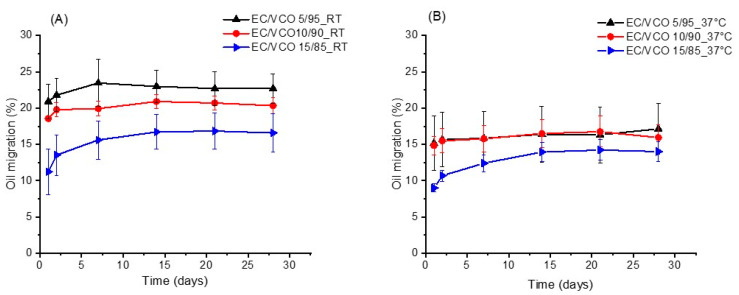
Oil migration from the EC/VCO oleogels to the filter paper for 28 days. Temperature conditions: (**A**) room temperature and (**B**) 37 °C.

The ability of the EC/VCO-based oleogels to effectively retain liquid oil over time was evaluated as a function of time (Figure 4). During the first few days (1–3 days), the liquid oil was quickly released from the gel network; from day 7 up to day 28, the oil migration from all samples showed a tendency to stabilize. Moreover, the samples with higher VCO content, namely EC/VCO 5/95 and EC/VCO 10/90, have similar oil migration, while EC/VCO 15/85 showed lower oil migration capability. The higher OBC for EC/VCO 15/85 in both conditions, RT (88.5 ±1.4%) and 37 °C (87.8 ± 0.9%), can explain those results, as the gel network entrapped the VCO more efficiently into the structure. Similar studies on oleogels made with sunflower oil suggested that the strength of the network would be useful for avoiding the oil release from the oleogel [2]. The EC/VCO’s oil migration behavior and the moisturizing skin effects of the VCO [45] suggest that those oleogels may be helpful for biomedical applications or even as a delivery system, especially if a liposoluble bioactive agent is considered.

### 3.5. Antioxidant Activity

**Figure 5:**
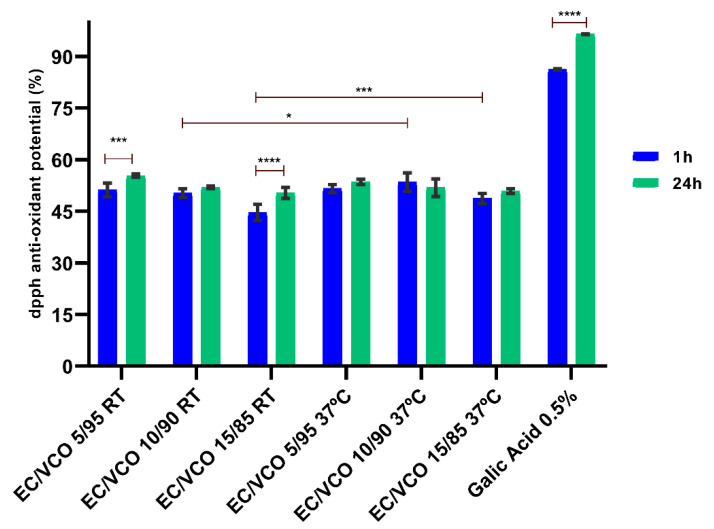
Antioxidant activity of the EC/VCO-based oleogels compared with gallic acid (**** *p* < 0.0001, *** *p* < 0.001 and * *p* < 0.05).

The antioxidant activity of the developed EC/VCO oleogels was evaluated using the DPPH radical scavenging assay [28]. Gallic acid, a naturally occurring polyphenol, was used as a positive control, since it presents a strong antioxidant and free radical scavenging ability [46]. The results in Figure 5 indicated that EC/VCO oleogels have high antioxidant activity (up to 57%), and this trend was maintained for up to 24 h. Additionally, the gallic acid (control) had the highest scavenging activity observed: 96%. From a general point of view, the statistical analysis did not reveal significant differences between the EC/VCO formulations and the effect of the molding temperature. Still, significant differences were observed for all formulations compared to the control (gallic acid). VCO is a powerful antioxidant product which can be used to fight against free radicals in the human body, and it helps to slow down the aging process [47,48]. This bioactivity is possibly associated with the VCO polyphenolic compound, as suggested by some earlier studies [49].

### 3.6. Stability Behavior

The prepared EC/VCO oleogels were subjected to stability testing to understand the short-term and long-term stability of the formulations in PBS and pH = 5 buffer solution for up to 28 days. It is predictable that, as time goes by, the oil migration from the samples to the medium would affect the samples’ integrity. However, considering the components’ hydrophobic nature (EC and VCO), we do not expect accelerated degradation. In fact, most of the formulations maintained their stability, and they have resistance in the studied media for up to 28 days. Furthermore, upon analysis of the samples’ weight loss after 28 days in the media (Table 3), it is possible to note that the weight loss of the oleogels decreased in both media with the increase in the EC content being the only significant difference registered (*p* < 0.0001) compared with any formulation with the EC/VCO 5/95_RT formulation at both analyzed pHs. As EC is known to degrade slowly in vivo [50], it is expected that the network formed between EC and VCO could allow modulation of the stability/degradation of the matrices. In addition, the achieved data agree with the observations of the OBC assay as the formulations with high OBC also showed lower weight loss, suggesting that the polymeric network also contributes to retaining the oil release and, in turn, enhancing their stability in the medium.

### 3.7. Thermal Analysis

**Figure 6:**
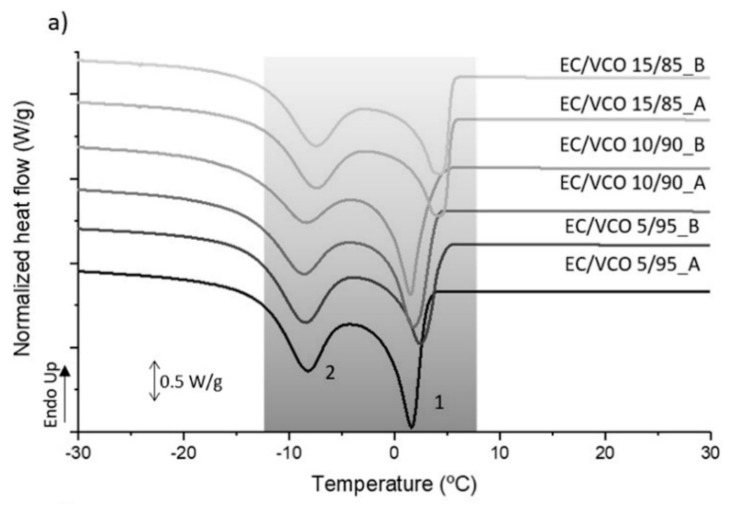

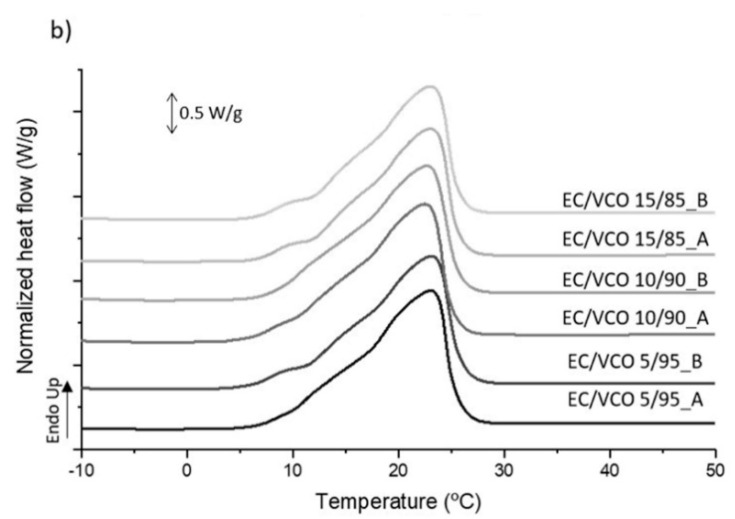
DSC thermograms obtained for EC/VCO oleogels using 10 °C/min^−1^; (**a**) crystallization process and (**b**) melting process.

The thermal properties of the oleogels were analyzed by DSC in the crystallization and considering the second melting processes. These experiments were conducted by dissolving the EC/VCO oleogel mixtures in the first heating cycle up to 160 °C to erase the thermal story and subsequently cooling to form a gel, followed by a second heating cycle for comparison of the different processed conditions. Figure 6 presents the thermograms of EC/VCO oleogels and the results for the main peaks, maximum crystallization temperature (T_c_ (peak 1, high temperature), T_c_ (peak 2, lower temperature) and T_m_)) and the variation of enthalpy (crystallization ΔH_c_ and melting ΔH_m_) are presented in Table S1 in Supplementary Information. The thermograms of the main used constituents in the oleogel preparation are shown in Figure S2, where the EC of medium molecular weight reveals the glass transition at 116 °C (see inset graph) and an exothermic event around 200 °C, which, according to the literature, might be associated with oxidative degradation [51,52]. The VCO shows a major melting peak at 23.41 °C; however, it is possible to observe a smaller shoulder at a lower temperature (around 15 °C) that might be attributed to the co-melting of triacylglycerol (TAG) molecules with different melting temperatures [53]. The DSC heating process (Figure 6b) of the EC/VCO oleogels has similar behavior, but they showed a small shift on Tm. Although the TAG molecules remain in the EC/VCO system, the interactions between VCO and EC molecules, in different percentages, could be responsible for that thermal behavior. In the crystallization process, the VCO displayed two exothermic peaks at 2.34 °C and −6.20 °C. The different temperatures obtained for the crystallization temperature could be explained by the co-crystallization of TAG molecules [53], where VCO, like other oils, consists of a complex mixture of triglycerides. When subjected to the crystallization conditions from the molten state, the oils pass through the nucleation, activation, crystal growth, and crystal lattice stage like polymers [54]. In general, during the cooling process of the oleogels (Figure 6a), a slight shift in the crystallization temperature of peak 1 (high-temperature peak), was observed with increased EC gelator content in the system; however, no correlation with the oleogel temperature storage (RT or 37 °C) was found. Previous studies on EC oleogels [41] suggested that the increasing EC content interferes with the lauric acid crystallization due to steric hindrance, thus shifting the crystallization peaks to a lower temperature. In addition, a small decrease in the enthalpy of crystallization from 80.43 to 70.65 J/g (Table 3, samples RT) and from 85.58 to 72.44 J/g (samples 37 °C) was noted. However, no significant differences in the enthalpy were observed for samples with the same composition produced at different cooling temperatures. Furthermore, a depletion of the number of free VCO fatty acids inside the EC/VCO system due to hydrogen bonds formation (oil binding discussion, Figure 3B) could also contribute to the observed findings.

## 4. Conclusions

This study demonstrated that oleogels could be structured by combining EC and VCO, representing a promising research avenue. The findings evidenced the EC and VCO’s good compatibility. Additionally, they showed that by varying the EC and VCO content and the molding temperature, it is possible to obtain oleogels with a viscoelastic nature, adequate structural stability, modulable stiffness, high oil-binding capability, suitable antioxidant activity, and good thermal stability. Furthermore, the characterization of those oleogels demonstrated that an increase in EC content had a key role in the oil–polymer interactions, and consequently in the network features, oleogel strength, and stability. Based on the features of the developed EC/VCO-based architectures, some applications can be foreseen; for instance, they could be useful as structured lipid systems to transport bioactive compounds, as well as in the cosmetics, biomedical, and manufacturing industries. Furthermore, based on the application, it is foreseen that other melt-based technologies might be employed to the oleogel systems, providing new shapes and properties to the developed materials.

## Figures and Tables

**Table 1:** Experimental conditions of the EC/VCO-based oleogels.

Identification	Ethylcellulose Content (%)	Virgin Coconut Oil Content (%)	Molding Temperature
EC/VCO 5/95_RT	5	95	RT
EC/VCO 5/95_37 °C	5	95	37 °C
EC/VCO 10/90_RT	10	90	RT
EC/VCO 10/90_37 °C	10	90	37 °C
EC/VCO 15/85_RT	15	85	RT
EC/VCO 15/85_37 °C	15	85	37 °C

**Table 2:** Storage (G′), loss (G″), and loss factor (δ) moduli at 1 Hz frequency and 25 °C.

Composition	G′ (kPa)	G″ (kPa)	tanδ
EC/VCO 5/95_RT	1.13 ± 0.12	7.46 ± 0.68	6.60
EC/VCO 5/95_37 °C	0.83 ± 0.05	0.59 ± 0.30	0.71
EC/VCO 10/90_RT	127.0 ± 22.62	19.55 ± 5.26	0.15
EC/VCO 10/90_37 °C	199.20 ± 92.35	33.03 ± 13.17	0.17
EC/VCO 15/85_RT	106.45 ± 0.35	16.37 ± 0.60	0.15
EC/VCO 15/85_37 °C	279.65 ± 41.79	50.90 ± 8.62	0.18

**Table 3:** Weight loss of the oleogel samples after immersion in PBS and pH = 5 up to 28 days.

Sample	Weight Loss (%)	
	PBS	pH = 5
EC/VCO 5/95_RT	73.9 ± 14.8	61.1 ± 6.4
EC/VCO 5/95_37 °C	*	*
EC/VCO 10/90_RT	23.9 ± 2.9	18.1 ± 2.9
EC/VCO 10/90_37 °C	13.20 ± 4.5	21.06 ± 5.9
EC/VCO 15/85_RT	16.20 ± 6.2	11.12 ± 1.9
EC/VCO 15/85_37 °C	23.66 ± 1.8	19.18 ± 4.0

* The sample loses the shape after 14 days, and there is no stability to continue the experiment.

## Data Availability

Research data are not shared.

## Supplementary Materials

The following supporting information can be downloaded https://www.mdpi.com/article/10.3390/polym14122473/s1, Figure S1: FTIR spectra of ethylcellulose, virgin coconut oil and developed samples; Figure S2: DSC thermograms of the main constituents based on VCO and EC; Table S1: Thermal parameters of the oleogels.

## References

[1] Pușcaș A., Mureșan V., Socaciu C., Muste S. (2020). Oleogels in Food: A Review of Current and Potential Applications. Foods.

[2] Bascuas S., Salvador A., Hernando I., Quiles A. (2020). Designing Hydrocolloid-Based Oleogels with High Physical, Chemical, and Structural Stability. Front. Sustain. Food Syst..

[3] Davidovich-Pinhas M., Barbut S., Ag M. (2016). Development, Characterization, and Utilization of Food-Grade Polymer Oleogels. Annu. Rev. Food Sci. Technol..

[4] Ismail N.A., Amin K.A.M., Razali M.H. (2018). Mechanical and Antibacterial Activities Study of Gellan Gum/Virgin Coconut Oil Film Embedded Norfloxacin. IOP Conference Series: Materials Science and Engineering.

[5] Singh A., Auzanneau F.I., Rogers M.A. (2017). Advances in edible oleogel technologies–A decade in review. Food Res. Int..

[6] Bascuas S., Morell P., Hernando I., Quiles A. (2021). Recent trends in oil structuring using hydrocolloids. Food Hydrocoll..

[7] Pinto T.C., Martins A.J., Pastrana L., Pereira M.C., Cerqueira M.A. (2021). Oleogel-Based Systems for the Delivery of Bioactive Compounds in Foods. Gels.

[8] Martins A.J., Vicente A.A., Pastrana L.M., Cerqueira M.A. (2020). Oleogels for development of health-promoting food products. Food Sci. Hum. Wellness.

[9] Rogers M.A., Wright A.J., Marangoni A.G. (2009). Oil organogels: The fat of the future?. Soft Matter.

[10] Giacintucci V., Di Mattia C.D., Sacchetti G., Flamminii F., Gravelle A.J., Baylis B., Dutcher J.R., Marangoni A.G., Pittia P. (2018). Ethylcellulose oleogels with extra virgin olive oil: The role of oil minor components on microstructure and mechanical strength. Food Hydrocoll..

[11] Gravelle A.J., Barbut S., Marangoni A.G. (2013). Fractionation of ethylcellulose oleogels during setting. Food Funct..

[12] Wasilewska K., Winnicka K. (2019). Ethylcellulose—A Pharmaceutical Excipient with Multidirectional Application in Drug Dosage Forms Development. Materials.

[13] Gravelle A.J., Davidovich-Pinhas M., Zetzl A.K., Barbut S., Marangoni A.G. (2016). Influence of solvent quality on the mechanical strength of ethylcellulose oleogels. Carbohydr. Polym..

[14] Davidovich-Pinhas M., Gravelle A.J., Barbut S., Marangoni A.G. (2015). Temperature effects on the gelation of ethylcellulose oleogels. Food Hydrocoll..

[15] Roopan S.M. (2016). An Overview of Phytoconstituents, Biotechnological Applications, and Nutritive Aspects of Coconut (*Cocos nucifera*). Appl. Biochem. Biotechnol..

[16] Marina A.M., Che Man Y.B., Amin I. (2009). Virgin coconut oil: Emerging functional food oil. Trends Food Sci. Technol..

[17] Khor Y.P., Koh S.P., Long K., Long S., Ahmad S.Z., Tan C.P. (2014). A comparative study of the physicochemical properties of a virgin coconut oil emulsion and commercial food supplement emulsions. Molecules.

[18] Dayrit F. (2015). The Properties of Lauric Acid and Their Significance in Coconut Oil. J. Am. Oil Chem. Soc..

[19] Intahphuak S., Khonsung P., Panthong A. (2010). Anti-inflammatory, analgesic, and antipyretic activities of virgin coconut oil. Pharm. Biol..

[20] Pandiselvam R., Ramarathinam M., Beegum S., Mathew A. (2019). Virgin Coconut Oil infused healthy cosmetics. Indian Coconut J..

[21] Wróblewska M., Szymańska E., Szekalska M., Winnicka K. (2020). Different Types of Gel Carriers as Metronidazole Delivery Systems to the Oral Mucosa. Polymers.

[22] Silva S.S., Rodrigues L.C., Fernandes E.M., Gomes J.M., Vilas-Boas Â., Pirraco R.P., Reis R.L. (2020). Approach on chitosan/virgin coconut oil-based emulsion matrices as a platform to design superabsorbent materials. Carbohydr. Polym..

[23] Ismail N.A., Mohamad S.F., Ibrahim M.A., Mat Amin K.A. (2014). Evaluation of Gellan Gum Film Containing Virgin Coconut Oil for Transparent Dressing Materials. Adv. Biomater..

[24] Tan S.Y., Wan Y.P.E., Marangoni A.G., Henry C.J. (2017). Effects of liquid oil vs. oleogel co-ingested with a carbohydrate-rich meal on human blood triglycerides, glucose, insulin and appetite. Food Funct..

[25] Tan S.Y., Peh E., Lau E., Marangoni A.G., Henry C.J. (2017). Physical Form of Dietary Fat Alters Postprandial Substrate Utilization and Glycemic Response in Healthy Chinese Men. J. Nutr..

[26] Ahmadi P., Tabibiazar M., Roufegarinejad L., Babazadeh A. (2020). Development of behenic acid-ethyl cellulose oleogel stabilized Pickering emulsions as low calorie fat replacer. Int. J. Biol. Macromol..

[27] Dawson R., Elliot D., Elliot W., Jones K., Wood E.J. (1987). Data for Biochemical Research. Biochemical Education.

[28] Kedare S.B., Singh R.P. (2011). Genesis and development of DPPH method of antioxidant assay. J. Food Sci. Technol..

[29] Laredo T., Barbut S., Marangoni A.G. (2011). Molecular interactions of polymer oleogelation. Soft Matter..

[30] Gravelle A.J., Barbut S., Marangoni A.G. (2012). Ethylcellulose oleogels: Manufacturing considerations and effects of oil oxidation. Food Res. Int..

[31] Ye X., Li P., Lo Y.M., Fu H., Cao Y. (2019). Development of Novel Shortenings Structured by Ethylcellulose Oleogels. J. Food Sci..

[32] Yılmaz E., Uslu E.K., Toksöz B. (2020). Structure, Rheological and Sensory Properties of Some Animal Wax Based Oleogels. J. Oleo Sci..

[33] Patel A.R., Babaahmadi M., Lesaffer A., Dewettinck K. (2015). Rheological Profiling of Organogels Prepared at Critical Gelling Concentrations of Natural Waxes in a Triacylglycerol Solvent. J. Agric. Food Chem..

[34] Rodríguez-Hernández A.K., Pérez-Martínez J.D., Gallegos-Infante J.A., Toro-Vazquez J.F., Ornelas-Paz J.J. (2021). Rheological properties of ethyl cellulose-monoglyceride-candelilla wax oleogel vis-a-vis edible shortenings. Carbohydr. Polym..

[35] Kwon U.H., Chang Y.H. (2022). Rheological and Physicochemical Properties of Oleogel with Esterified Rice Flour and Its Suitability as a Fat Replacer. Foods.

[36] Luo S.Z., Hu X.F., Jia Y.J., Pan L.H., Zheng Z., Zhao Y.Y., Mu D.D., Zhong X.Y., Jiang S.T. (2019). Camellia oil-based oleogels structuring with tea polyphenol-palmitate particles and citrus pectin by emulsion-templated method: Preparation, characterization and potential application. Food Hydrocoll..

[37] Meng Z., Qi K., Guo Y., Wang Y., Liu Y. (2018). Effects of thickening agents on the formation and properties of edible oleogels based on hydroxypropyl methyl cellulose. Food Chem..

[38] Abdollahi M., Goli S.A.H., Soltanizadeh N. (2020). Physicochemical Properties of Foam-Templated Oleogel Based on Gelatin and Xanthan Gum. Eur. J. Lipid Sci Technol..

[39] Pușcaș A., Mureșan V., Muste S. (2021). Application of Analytical Methods for the Comprehensive Analysis of Oleogels-A Review. Polymers.

[40] Szymańska I., Żbikowska A., Kowalska M. (2020). Physical stability of model emulsions based on ethyl cellulose oleogels. Int. Agrophys..

[41] Haj Eisa A., Laufer S., Rosen-Kligvasser J., Davidovich-Pinhas M. (2020). Stabilization of Ethyl-Cellulose Oleogel Network Using Lauric Acid. Eur. J. Lipid Sci. Technol..

[42] Trivedi M., Branton A., Trivedi D., Nayak G., Mishra R., Jana S. (2015). Characterization of Physicochemical and Thermal Properties of Biofield Treated Ethyl Cellulose and Methyl Cellulose. Int. J. Biomed. Mater. Res..

[43] Rohman A., Che Man Y.B., Ismail A., Hashim P. (2010). Application of FTIR Spectroscopy for the Determination of Virgin Coconut Oil in Binary Mixtures with Olive Oil and Palm Oil. J. Am. Oil Chem. Soc..

[44] Davidovich-Pinhas M., Barbut S., Marangoni A.G. (2015). The gelation of oil using ethyl cellulose. Carbohydr. Polym..

[45] Varma S.R., Sivaprakasam T.O., Arumugam I., Dilip N., Raghuraman M., Pavan K.B., Rafiq M., Paramesh R. (2019). In vitro anti-inflammatory and skin protective properties of Virgin coconut oil. J. Tradit. Complement. Med..

[46] Badhani B., Sharma N., Kakkar R. (2015). Gallic acid: A versatile antioxidant with promising therapeutic and industrial applications. RSC Adv..

[47] Narayanankutty A., Illam S.P., Raghavamenon A.C. (2018). Health impacts of different edible oils prepared from coconut (Cocos nucifera): A comprehensive review. Trends Food Sci. Technol..

[48] Ng Y.J., Tham P.E., Khoo K.S., Cheng C.K., Chew K.W., Show P.L. (2021). A comprehensive review on the techniques for coconut oil extraction and its application. Bioprocess Biosyst. Eng..

[49] Marina A.M., Man Y.B., Nazimah S.A., Amin I. (2009). Antioxidant capacity and phenolic acids of virgin coconut oil. Int. J. Food Sci. Nutr..

[50] Elçin A.E. (2006). In Vitro and In Vivo Degradation of Oxidized Acetyl- and Ethyl-Cellulose Sponges. Artif. Cells Blood Substit. Biotechnol..

[51] Lai H.L., Pitt K., Craig D.Q.M. (2010). Characterisation of the thermal properties of ethylcellulose using differential scanning and quasi-isothermal calorimetric approaches. Int. J. Pharm..

[52] Dubernet C., Rouland J., Benoit J. (1990). Comparative study of two ethylcellulose forms (raw material and microspheres) carried out through thermal analysis. Int. J. Pharm..

[53] Marikkar J.M.N. (2019). Differential Scanning Calorimetric Analysis of Virgin Coconut Oil, Palm Olein, and their Adulterated Blends. CORD.

[54] Srivastava Y., Semwal A.D., Sajeevkumar V.A., Sharma G.K. (2017). Melting, crystallization and storage stability of virgin coconut oil and its blends by differential scanning calorimetry (DSC) and Fourier transform infrared spectroscopy (FTIR). J. Food Sci. Technol..

