# Supplementary material for: Tailoring Natural-Based Oleogels Combining Ethylcellulose and Virgin Coconut Oil
Source: Polymers (Basel). 2022 Jun 17;14(12):2473. doi: 10.3390/polym14122473 (PMC9230444; doi:10.3390/polym14122473)
Supplement: Supplementary file 1 [file polymers-14-02473-s001.zip › polymers-1755099-supplementary.pdf]

polymers

MDPI

Supplementary information

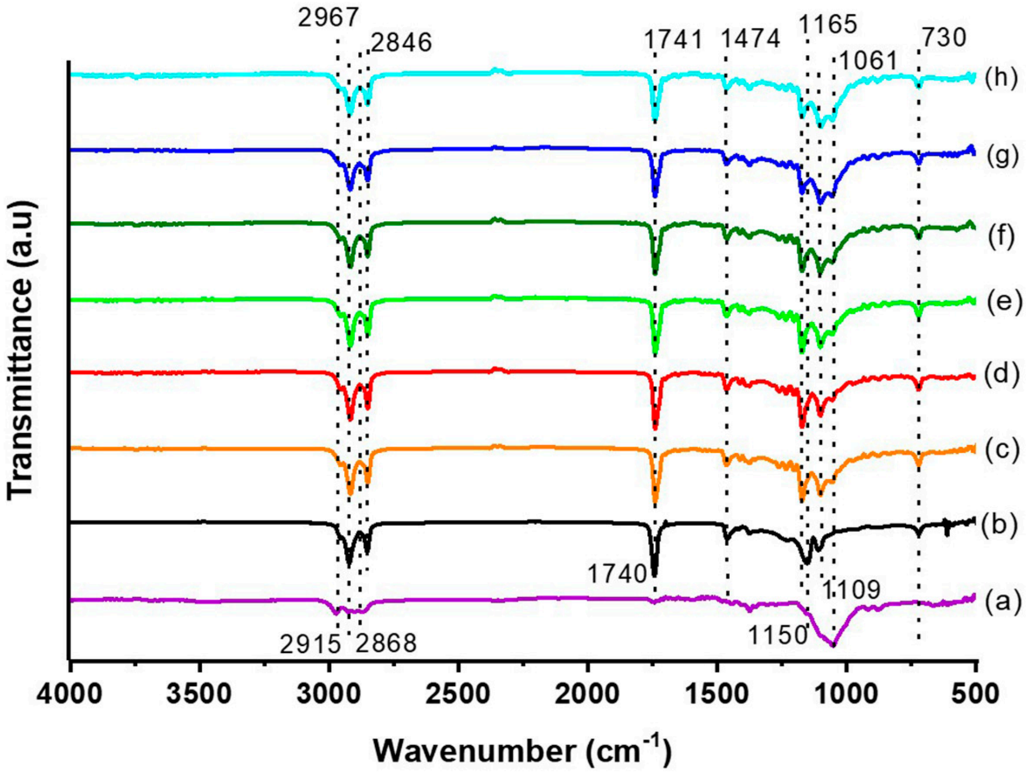

Figure S1: FTIR spectra of ethylcellulose (a), virgin coconut oil (b), EC/VCO 5/95_37°C (c), EC/VCO 5/95_RT (d), EC/VCO 10/90_37°C (e), EC/VCO 10/90_RT (f), EC/VCO 15/85_37°C (g) and EC/VCO 15/85_RT (h).

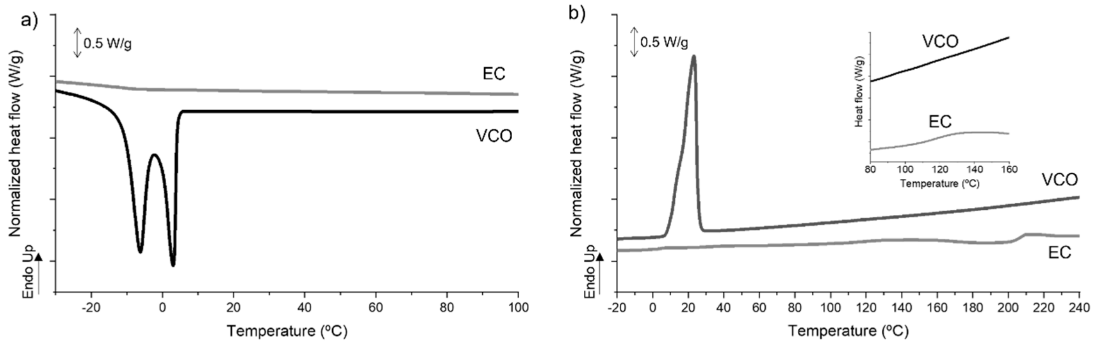

Figure S2: DSC thermograms of the main constituents based on VCO and EC used for the oleogel production during: (a) second heating cycle and (b) cooling process.

Table S1. Thermal parameters of the oleogels (temperature of crystallization, Tc; and enthalpy of crystallization ΔH_c; melting temperature, Tm; melting enthalpy ΔH_m).

	**Crystallization**			**Melting**	
**Condition**	**Peak 1 Tc (°C)**	**Peak 2 Tc (°C)**	**ΔHc (J/g)**	**Tm (°C)**	**ΔHm (J/g)**
VCO	2.34 ± 0.24	−6.20 ± 0.22	83.41 ± 5.88	23.41 ± 0.15	92.21 ± 5.19
EC/VCO 5/95_RT	1.69 ± 0.19	−8.25 ± 0.16	80.43 ± 1.40	22.97 ± 0.24	92.19 ± 1.51
EC/VCO 5/95_37°C	2.58 ± 0.87	−8.11 ± 0.64	85.58 ± 1.74	23.43 ± 0.59	95.53 ± 1.14
EC/VCO 10/90_RT	1.71 ± 0.15	−8.63 ± 0.11	75.97 ± 0.40	22.91 ± 0.45	89.07 ± 0.83
EC/VCO 10/90_37°C	1.32 ± 0.36	−8.51 ± 0.01	75.09 ± 1.53	21.96 ± 1.33	87.79 ± 1.07
EC/VCO 15/85_RT	2.26 ± 0.48	−8.60 ± 0.29	70.65 ± 1.56	21.56 ± 1.49	83.04 ± 1.99
EC/VCO 15/85_37°C	3.27 ± 1.10	−7.49 ± 0.55	72.44 ± 2.71	22.96 ± 1.49	83.95 ± 1.43
